# Biomedical Interpenetrated Hydrogels Fabricated via Quaternary Ammonium Chitosan and Dopamine-Conjugated Gelatin Integrated with Genipin and Epigallocatechin Gallate

**DOI:** 10.3390/gels12010067

**Published:** 2026-01-11

**Authors:** Ling Wang, Shuxin Hu, Zheng Wei, Peng Ding, Yaling Deng, Yanting Han, Yanfang Sun, Guohua Jiang, Lei Nie

**Affiliations:** 1 College of Life Sciences, Xinyang Normal University (XYNU), Xinyang 464000, China; wangling@xynu.edu.cn (L.W.); hushuxin060907@163.com (S.H.); weizheng20050815@163.com (Z.W.); dingzhiyu120@163.com (P.D.); hanyt@xynu.edu.cn (Y.H.); 2 College of Intelligent Science and Control Engineering, Jinling Institute of Technology, Nanjing 211169, China; yalingdeng@jit.edu.cn; 3 College of Life Sciences and Medicine, Zhejiang Sci-Tech University, Hangzhou 310018, China; katherineyfs@zstu.edu.cn; 4 School of Materials Science and Engineering, Zhejiang Sci-Tech University, Hangzhou 310018, China; ghjiang_cn@zstu.edu.cn; 5 International Scientific and Technological Cooperation Base of Intelligent Biomaterials and Functional Fibers, Zhejiang Sci-Tech University, Hangzhou 310018, China

**Keywords:** chitosan, gelatin, genipin, EGCG, hydrogels

## Abstract

Multifunctional hydrogels with an interpenetrated network structure have shown great potential for biomedical and tissue-regeneration applications. In this work, the biomedical hydrogel was fabricated with an interpenetrated network based on dopamine grafted gelatin (DA-Gel), and genipin crosslinked quaternary ammonium chitosan (QCS), incorporating epigallocatechin gallate (EGCG). The EDC/NHS and Schiff-base bond connections occurred in the hydrogels, as confirmed by Fourier-transform infrared (FT-IR) analysis. The properties of the fabricated hydrogels, including microstructure, degradation rate, adhesive strength, mechanical strength, and rheological behavior, can be regulated by adjusting the DA-Gel/QCS ratio or by using different crosslinking approaches. In addition, the fabricated hydrogels exhibited self-healing properties and strong adhesion to various materials and organs. Furthermore, the hydrogels performed good antibacterial activity against the typical bacteria, *Escherichia coli* and *Staphylococcus aureus*. EGCG encapsulated hydrogels displayed excellent antioxidant activities and good hemocompatibility. The hydrogels also demonstrated excellent cytocompatibility and good cell migration ability. The above results provide a facile approach to fabricate the biomedical hydrogels with a regulated network structure and multifunctional characteristics with potential in biomedical applications.

## 1. Introduction

Hydrogels are three-dimensional (3D) polymeric networks capable of retaining large amounts of water while maintaining structural integrity [1]. Owing to their high water content, tunable mechanical properties, and inherent biocompatibility, hydrogels have been extensively investigated for various biomedical applications, including wound dressings, drug delivery systems, tissue engineering scaffolds, and biosensors [2,3]. However, despite these advantages, conventional hydrogels often suffer from several intrinsic limitations, such as weak mechanical strength, poor structural stability under physiological conditions, and limited biological functionality, including antibacterial activity, antioxidant capacity and biocompatibility [4]. To overcome these challenges, recent research has focused on designing hybrid or composite hydrogels to achieve improved mechanical robustness and biological performance. In addition, the hydrogels possess good antibacterial activity and antioxidant capacity, which facilitates their biomedical applications, such as accelerating the bacterial-infected skin tissue regeneration [5,6,7].

The physicochemical properties of hydrogels could be manipulated via adjusting the molecular networks, such as by establishing noncovalent interactions or covalent bonds [8,9,10]. An interpenetrating polymer network (IPN) is a unique class of hybrid polymeric materials in which two or more networks are physically interlaced but not covalently bonded to each other [11]. The combination of independent networks allows the resulting hydrogels to integrate the advantages of each polymer while compensating for their individual shortcomings [12]. Through the synergistic structure, IPN hydrogels often exhibit significantly enhanced toughness, elasticity, and durability [13]. Moreover, a dual-network architecture provides opportunities for integrating multiple functions into a single hydrogel platform, such as adhesion, antibacterial activity, and antioxidant effects [14]. Therefore, the IPN design strategy represents a promising pathway for developing next-generation biomedical hydrogels capable of meeting the complex demands of tissue repair, regeneration, and therapeutic delivery.

Building upon this concept, biopolymer-based hydrogels have gained increasing attention due to their biocompatibility and functional diversity. Among them, chitosan-based hydrogels, obtained from shrimp and crab, have attracted extensive attention because of their good biocompatibility, blood compatibility, biodegradability, and inherent hemostatic and antibacterial properties [15]. However, chitosan’s poor solubility under neutral conditions limits its biomedical applications. The introduction of quaternary ammonium groups can effectively improve its solubility, thus broadening its application range [16]. In addition, quaternary ammonium chitosan (QCS) has strong tissue-adhesive properties owing to its large number of cations, which can interact electrostatically with negatively charged cell membranes or tissue surfaces [17]. Therefore, QCS provides both structural stability and antibacterial functionality, making it a suitable component for the construction of multifunctional hydrogels. In our previous results, the QCS polymer could be crosslinked with genipin and still exhibited excellent antibacterial activity and cytocompatibility. Furthermore, the functional hydrogels with an IPN structure could be facilely fabricated by introducing the second polymer chain, such as hyaluronic acid, chondroitin sulfate, etc. [18,19,20].

Gelatin, a collagen-derived natural polymer, is another biocompatible and biodegradable material widely used in tissue engineering [21]. Compared with collagen, gelatin exhibits unique inherent properties, including an elastic nature, low antigenicity, ease of chemical modification, and low cost and easy availability [22]. However, gelatin is well known to present poor mechanical properties owing to its highly hygroscopic nature under wet conditions. Depending on temperature, gelatin can undergo structural changes [23,24]. Therefore, it is appealing to develop a new class of gelatin-based tissue adhesives that are not only mechanically stable but also exhibit strong tissue adhesion. To address these limitations, the introduction of dopamine (DA) into gelatin has emerged as an efficient strategy to enhance its adhesion and functional performance. Owing to its close structural resemblance to 3,4-dihydroxyl-L-phenylalanine (DOPA), the demonstrated responsible group for the wet adhesion mechanism in mussels, dopamine has been used to fabricate biomimetic materials with significant adhesive strength [25]. Under oxidation, an activated catechol group of dopamine loses two electrons and two protons, resulting in a highly reactive quinone that reacts with thiol and amine groups via Michael-type addition or Schiff-base formation, thereby forming strong covalent bonds with biological tissues [26]. Consequently, the conjugation of DA to gelatin (DA-Gel) can impart enhanced adhesion and antioxidative properties, offering potential for bioadhesive applications. Meanwhile, the high biodegradability of gelatin results from its enzymatic digestion or its high solubility under physiological conditions, which prevents its further application as a scaffold in tissue engineering [27]. To address this issue, one crosslinker of common interest is genipin [28]. Genipin, a natural product abundantly present in gardenia fruits, has been shown to be 10,000 times less toxic than glutaraldehyde (GA, another crosslinking reagent) [29]. The crosslinking between gelatin and genipin involves a rapid nucleophilic ring-opening followed by a slower substitution reaction forming stable covalent bonds, which makes GP a favorable crosslinking agent for biomedical use [28].

**Scheme 1:**
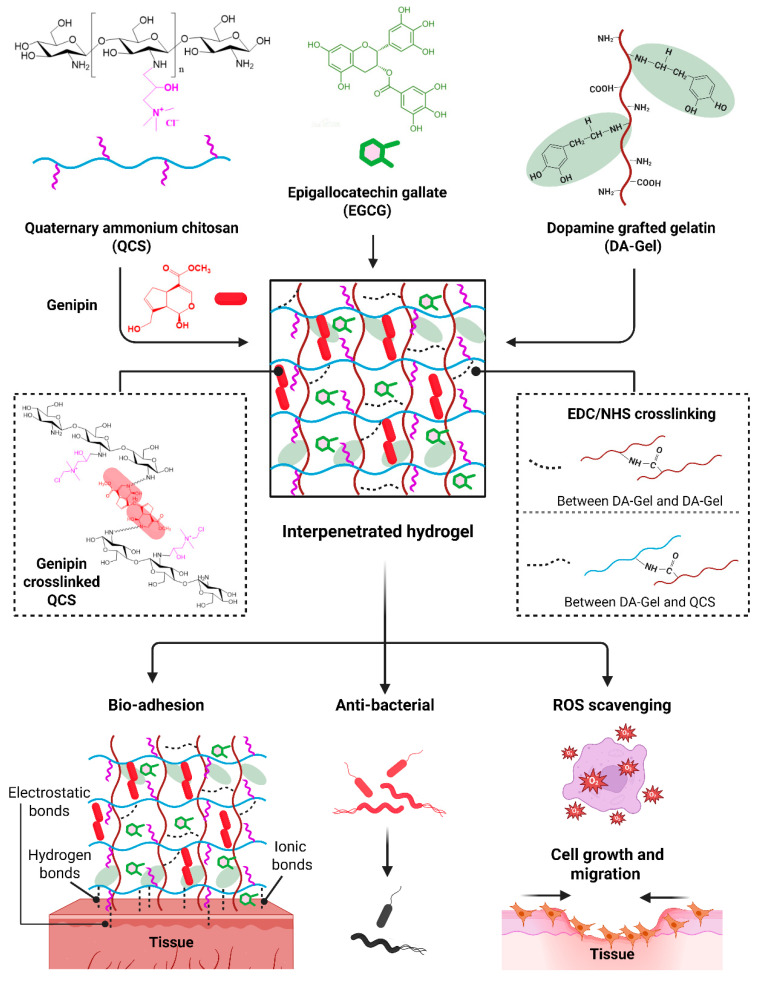
Schematic diagram of fabricating functional hydrogels via genipin crosslinked quaternary ammonium chitosan (QCS) and dopamine grafted gelatin (DA-Gel), incorporating epigallocatechin gallate (EGCG). The interpenetrated network was formed by interpenetrating EDC/NHS crosslinked DA-Gel into genipin crosslinked QCS. The fabricated hydrogels possess bioadhesive, antibacterial, ROS-scavenging, cell-growth, and pro-migration abilities for biomedical applications.

Epigallocatechin gallate (EGCG), the most abundant catechin in green tea, has been introduced into hydrogel systems as a multifunctional natural compound [30]. This catechin has attracted much interest owing to its numerous health benefits, including antioxidant, anti-inflammatory, and anti-tumor [31]. It is often used as a cross-linking agent for hydrogels through hydrogen bonding and π-π interactions. The incorporation of EGCG into polymer networks can enhance both the physicochemical stability and biological activity of hydrogels. Therefore, in this study, we fabricated a novel biomedical interpenetrated hydrogel system composed of quaternary ammonium chitosan (QCS) and dopamine-conjugated gelatin (DA-Gel), integrated with genipin (GP) and epigallocatechin gallate (EGCG), to achieve a multifunctional hydrogel (Scheme 1). In the system, QCS and DA-Gel integration can synergistically provide antibacterial and adhesive properties, respectively. The incorporation of GP as a biocompatible crosslinker and EGCG as a multifunctional component further enhances network stability, antioxidative capacity, and biocompatibility. After successful synthesis, the physicochemical, rheological, mechanical, adhesive, antibacterial, and antioxidant properties of the resulting hydrogel were systematically investigated.

## 2. Results and Discussion

### 2.1. Synthesis of QCS and Gel-DA

**Figure 1:**
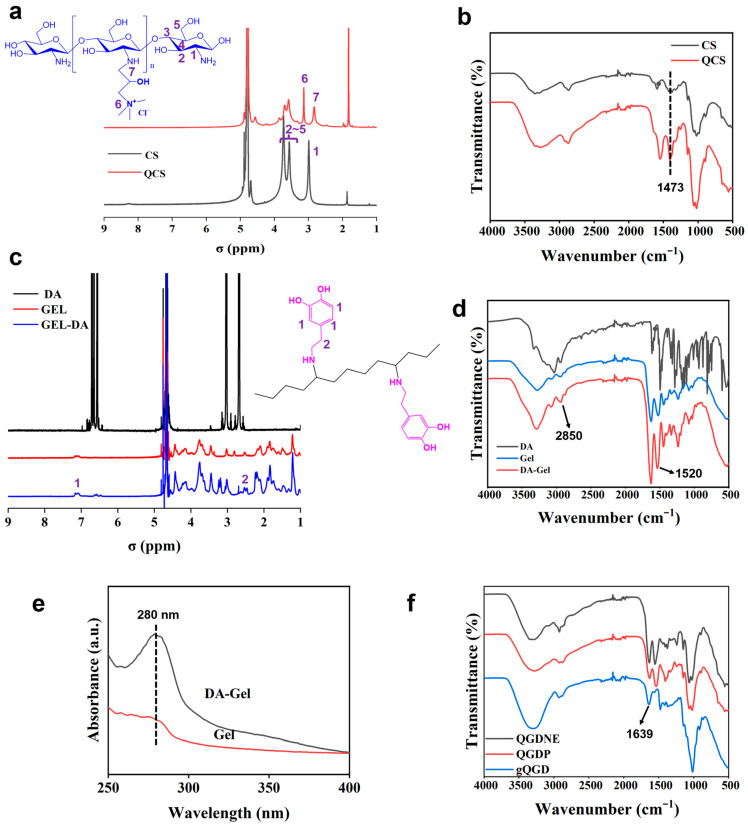
(**a**) ^1^H NMR spectra and (**b**) FT-IR spectra of CS and QCS. (**c**) ^1^H NMR spectra and (**d**) FT-IR spectra of dopamine, gelatin, and Gel-DA. (**e**) UV-vis absorbance spectra of gelatin and Gel-DA. (**f**) FT-IR spectra of different hydrogels, including QCDP, QGDNE, and gQGD hydrogels.

The polymer, quaternary ammonium chitosan (QCS), could be facilely synthesized by grafting quaternary ammonium groups onto chitosan [18,32]. ^1^H NMR and FT-IR were used to confirm the successful synthesis of QCS (Figure 1a,b). According to the ^1^H NMR spectrum of CS (Figure 1a), the peaks at 3.07 ppm and 3.64 ppm were attributed to the 1H protons at positions 1, 2, 3, 4, and 5 of the CS, respectively. While in the spectrum of QCS, the signal peaks at 3.13 ppm and 2.82 ppm were observed, and such peaks mainly contributed to the methyl groups from the quaternary ammonium groups in the synthesized QCS (positions 1, 2). In addition, the substitution degree (SD) of the quaternary ammonium group was calculated according to the ratio between ∫(N^+^(CH_3_)_3_) and ∫(H_1_), and the calculated SD value in the synthesized QCS was around 4.12 + 0.35%. In addition, the peak at 1473 cm^−1^ from the FT-IR spectrum of QCS, which was attributed to the N^+^-C group, also confirmed the successful synthesis of QCS by grafting quaternary ammonium groups in chitosan (Figure 1b) [18]. On the other hand, dopamine-conjugated gelatin (Gel-DA) was synthesized by grafting dopamine groups onto gelatin through an EDC/NHS coupling [33,34]. According to the ^1^H NMR spectrum of Gel-DA (Figure 1c), a peak was detected at 7.2 ppm, responding to the catechol ring of dopamine, confirming the successful graft of dopamine on gelatin. Furthermore, in the FT-IR spectrum of Gel-DA (Figure 1d), the typical peaks appeared at 1520 cm^−1^ and 2850 cm^−1^, belonging to the dopamine, due to the vibration of C-H and the stretching of the C=C ring, compared to the FT-IR spectrum of gelatin, proving the successful synthesis of Gel-DA. In addition, the grafted dopamine to gelatin was also detected using a UV-vis spectrophotometer. The typical peak at 280 nm, belonging to the dopamine group, was observed in the UV-vis spectrum of Gel-DA (Figure 1e). The above results confirmed that QCS and Gel-DA were successfully synthesized in this work.

### 2.2. Fabrication of Hydrogels

**Figure 2:**
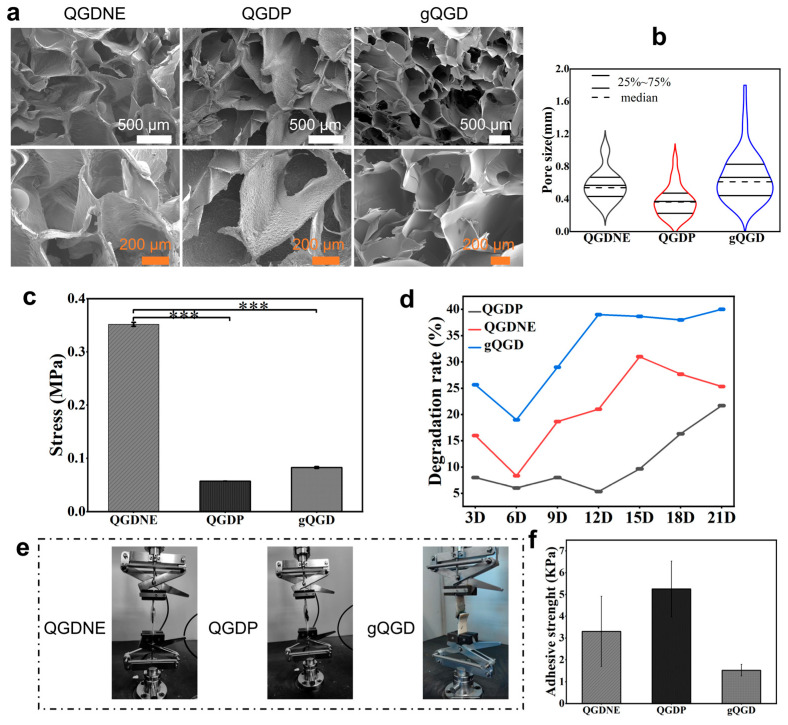
(**a**) SEM images of the fabricated hydrogels using different crosslinking strategies, including genipin crosslinking (QGDP hydrogel), EDC/NHS crosslinking (QGDNE hydrogel), and double genipin EDC/NHS crosslinking approaches (gQGD hydrogel). (**b**) Pore diameter distribution of hydrogels. The pore size was calculated using software according to five SEM images for each group. (**c**) The compressive stress of the fabricated hydrogels, *** *p* < 0.001. (**d**) The in vitro degradation profiles of the hydrogels were investigated in phosphate-buffered saline (PBS). (**e**) The adhesive strength of the hydrogels was measured using a lap-shear test, with wet pig skin used. (**f**) The measured adhesive strength of hydrogels.

Based on the synthesized QCS and Gel-DA polymers, three types of hydrogels with distinct crosslinking networks, prepared using different crosslinking approaches, were fabricated. The physicochemical and biological properties of hydrogels, including strength, adhesiveness, and self-healing ability, were strongly influenced by the crosslinking method [35,36]. In this paper, QGDP hydrogel was obtained via genipin crosslinking, QGDNE hydrogel via EDC/NHS crosslinking, and gQGD hydrogel via a double genipin-EDC/NHS crosslinking approach. From the FT-IR spectra of three kinds of fabricated hydrogels (Figure 1f), the typical peaks in the range of 1250–1350 cm^−1^, attributed to the amide II, were observed in QGDNE and gQGD hydrogels. Interestingly, the Schiff base bond peaks at 1639 cm^−1^ were also detected in the FT-IR spectra of hydrogels, revealing the potential of self-healing properties for all hydrogels, including QCDP, QGDNE, and gQGD. Next, the morphology and microstructure of the fabricated hydrogels were analyzed by SEM images (Figure 2a). From the SEM images, QCDP, QGDNE, and gQGD hydrogels all showed an interconnected porous microstructure. Based on the SEM images, the pore size distribution was statistically determined using ImageJ (Version 1.53), as shown in Figure 2b. The pore size of the hydrogels could be influenced by different crosslinking approaches. The interconnected microstructure was important for hydrogels in biomedical applications because it facilitates the transport of water and nutrient substances within the hydrogels [37,38].

The mechanical properties of all fabricated hydrogels were investigated. The compressive stresses were displayed in Figure 2c. It was clearly shown that the gQGD hydrogel with genipin and EDC/NHS crosslinking performed the highest tensile stress compared to QGDP and QGDNE hydrogels (Figure S1, Supporting Information). However, the QGDNE hydrogel displayed the highest compressive stress. The crosslinking method for fabricating hydrogels has a great effect on the mechanical properties and degradation rates [39]. As shown in Figure 2d, after 21 days of degradation, the hydrogels showed a mass reduction, with degradation rates exceeding 20% for all samples. Notably, gQGD maintained a higher degradation rate than the others, reaching up to 40%. This was because the gQGD had a less dense network structure that was more easily disrupted during the degradation test than those of other groups. During the 6–12-day interval, the degradation rates of gQGD and QGDNE increased rapidly. In addition to assessing the adhesion properties of the hydrogels on various substrates, the adhesion strength was also measured using a lap-shear test, and the wet pig skin was employed for evaluation. As presented in Figure 2e,f, the results indicated that QGDP demonstrated the strongest adhesion and the capacity of withstanding the greatest force.

### 2.3. Physicochemical Properties of gQGD Hydrogels

**Figure 3:**
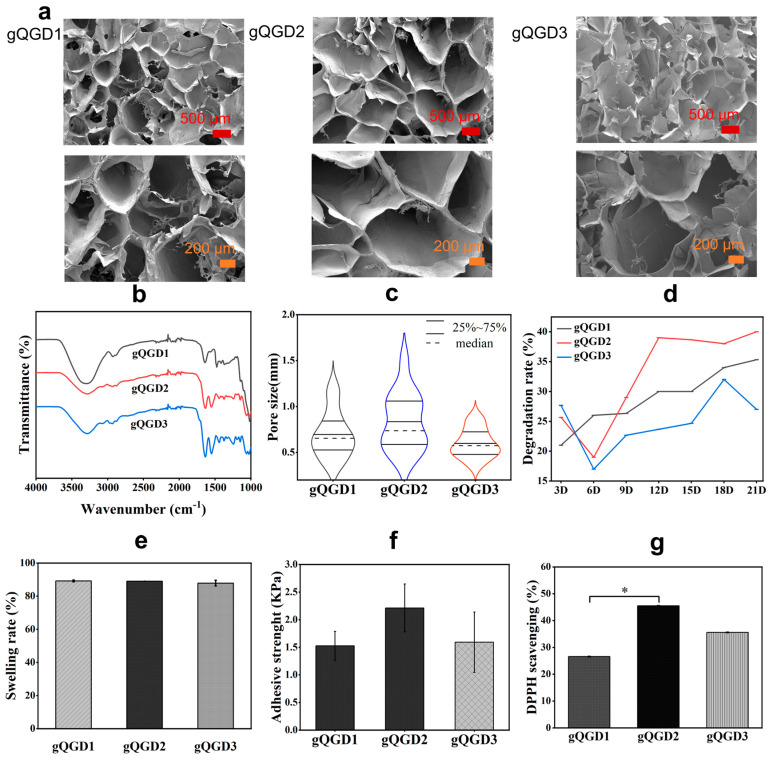
(**a**) SEM images (different magnifications) of the gQGD hydrogels fabricated using genipin and EDC/NHS crosslinking with different QCS/Gel-DA ratios. (**b**) FT-IR spectra of the gQGD hydrogels. (**c**) Pore size distribution of the gQGD hydrogels statistically calculated by ImageJ software (Version 1.53) according to SEM images. (**d**) The in vitro degradation profiles of gQGD hydrogels were investigated in phosphate-buffered saline (PBS). (**e**) The swelling rates of the gQGD hydrogels at the equilibrium state were measured in PBS. (**f**) The adhesive strength of the gQGD hydrogels was measured by the lap-shear test. (**g**) DPPH scavenging rates of the gQGD hydrogels, * *p* < 0.05.

The physicochemical properties of gQGD hydrogel with double genipin EDC/NHS crosslinking were investigated. With adjusting the QCS/Gel-DA ratios (7:3, 6:4, and 5:5), samples gQGD1, gQGD2, and gQGD3 hydrogels were fabricated. The porous architecture of gQGD hydrogels was observed using SEM images (Figure 3a). The fabricated gQGD hydrogels exhibited an interconnected pore structure, which was conducive to the absorption of water and the exchange of molecules, and enhanced the transportation of substances (such as nutrients, water, air, etc.), thereby promoting cell proliferation and accelerating wound healing [40,41]. The chemical interactions in gQGD hydrogels were investigated using FT-IR analysis. In the FT-IR spectrum of gQGD hydrogels (Figure 3b), the peak at 1473 cm^−1^, attributed to the N^+^-C group from QCS, was observed. Furthermore, the typical amide II and Schiff base bond peaks were detected, indicating genipin crosslinking in the gQGD hydrogels and EDC/NHS crosslinking in the gQGD hydrogels. Based on the obtained SEM images, pore size distribution was also determined using ImageJ software (Version 1.53) (Figure 3c). The gQGD2 hydrogel displayed a larger pore size compared to gQGD1 and gQGD3 hydrogels. Meanwhile, this feature also influenced its degradation rate. As shown in Figure 3d, after 21 days of degradation, the hydrogels showed a mass reduction, with degradation rates exceeding 25% for all samples. Notably, gQGD2 maintained a higher degradation rate than the others, reaching up to 40%. During the 6 to 12-day interval, the degradation rates of all hydrogels increased rapidly. Moreover, the mechanical properties of gQGD hydrogels were evaluated. According to the tensile stress–strain curves (Figure S1, Supporting Information), gQGD1 hydrogel with the highest QCS content performed the superior mechanical stress compared to gQGD2 and gQGD3 hydrogels. Next, the swelling properties of gQGD hydrogels were investigated, as shown in Figure 3e. The swelling ratios of gQGD1, gQGD2, and gQGD3 hydrogels after soaking in PBS solution for 24 h (37 °C and pH = 7.4) were 89.18%, 89.03%, and 87.88%, respectively. This result indicated that all three gQGD hydrogels had good swelling properties, effectively absorbing wound exudate, facilitating cell growth, and demonstrating potential for application as medical wound dressings [42,43].

The adhesion strength of gQGD hydrogels was measured using a porcine skin lap-shear test, and the measured adhesive strengths are shown in Figure 3f. The adhesion strength of gQGD2 was 2.0–2.5 KPa, which was higher than that of gQGD1 and gQGD3 (about 1.5 KPa), indicating that gQGD2 has stronger adhesion. Although the sample gQGD3 hydrogel has the highest Gel-DA content, the adhesion strength was lower than that of the gQGD2 hydrogel, indicating that the adhesive properties of the gQGD hydrogel were not only influenced by the dopamine groups, but also influenced by the genipin crosslinked QCS network. In our previous work, the genipin crosslinked QCS hydrogel also exhibited excellent adhesive strength, and the adhesion strength could be regulated by the crosslinking density [19]. Furthermore, the antioxidant capacity of gQGD hydrogels was also evaluated. As shown in Figure 3g, the DPPH Scavenging ratios of gQGD1, gQGD2, and gQGD3 hydrogels were 25%, 47%, and 36%, respectively. Among the three hydrogels, gQGD2 shows the best antioxidant activity.

### 2.4. Physicochemical Properties of gQGDE Hydrogels

**Figure 4:**
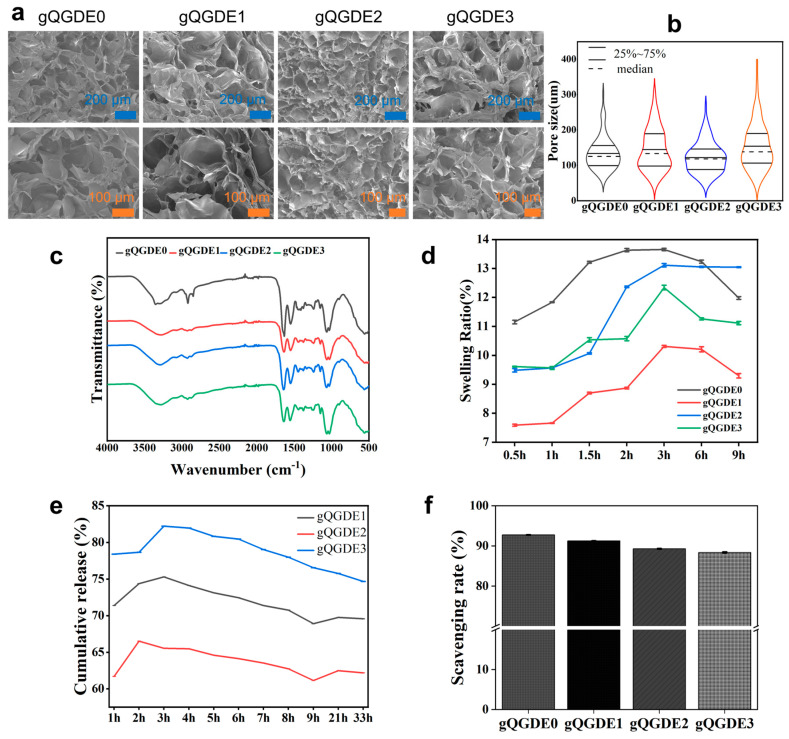
(**a**) SEM images of the gQGDE hydrogels with different EGCG content. (**b**) Pore size distribution of the gQGDE hydrogels. (**c**) FT-IR spectra of the gQGDE hydrogels. (**d**) The dynamic swelling rates of the gQGDE hydrogels in PBS. (**e**) The release profiles of EGCG from the gQGDE hydrogels in PBS. (**f**) DPPH scavenging rates of the gQGDE hydrogels.

By adjusting the EGCG content, gQGD, gQGDE0, gQGDE1, gQGDE2, and gQGDE3 hydrogels were fabricated. SEM images analysis of the hydrogels’ morphology and microstructure revealed a consistent interconnected pore network across all samples, as illustrated in Figure 4a. Using ImageJ software (Version 1.53), the pore size distribution was quantitatively assessed, showing no significant differences among the samples and indicating a uniform pore size distribution (Figure 4b). The FT-IR spectra of the gQGDE hydrogels were presented in Figure 4c, providing insights into their chemical composition and functional groups. The incorporation of EGCG into gQGDE hydrogels does not involve chemical interactions with the polymers, indicating that EGCG is mainly physically encapsulated within the hydrogel network.

According to the dynamic swelling test results (Figure 4d), gQGDE2 demonstrated the highest swelling ratio, with rapid expansion observed between 1.5 and 3 h, suggesting superior water absorption capacity. Conversely, gQGDE1 maintained a lower, more stable swelling ratio throughout the observation period, suggesting differences in crosslink density or polymer network structure. In addition, Figure 4e highlighted that the gQGDE3 hydrogel, which contained the highest concentration of EGCG (0.07 g), released the most active compound. The release rate of EGCG in gQGDE3 has reached 80% and was higher than that of Gqgde1 and gQGDE2. That was due to the higher pore size distribution in gQGDE3, which facilitates the EGCG release. Moreover, the release of all hydrogels is slow with an upward trend. This suggested an efficient delivery and potential therapeutic efficacy of EGCG from this formulation. Additionally, Figure 4f demonstrated that all gQGDE hydrogels possessed excellent antioxidant capacities, with activity levels exceeding 90%. It was interesting that there were no significant differences for all gQGDE hydrogels. In addition, the ABTS scavenging rates were also evaluated (Figure S2, Supporting Information), the results exhibited that gQGDE1, gQGDE2, and gQGDE3 hydrogels performed significantly higher ABTS scavenging rates than gQGDE0 hydrogel. This high antioxidant activity underscores their potential to reduce oxidative stress in wound environments, thereby promoting healing and tissue regeneration [44]. Overall, the comprehensive analysis indicated that the fabricated gQGDE hydrogels exhibited favorable structural, chemical, and functional properties suitable for biomedical applications, particularly in wound management.

### 2.5. Rheological Behavior Evaluation

**Figure 5:**
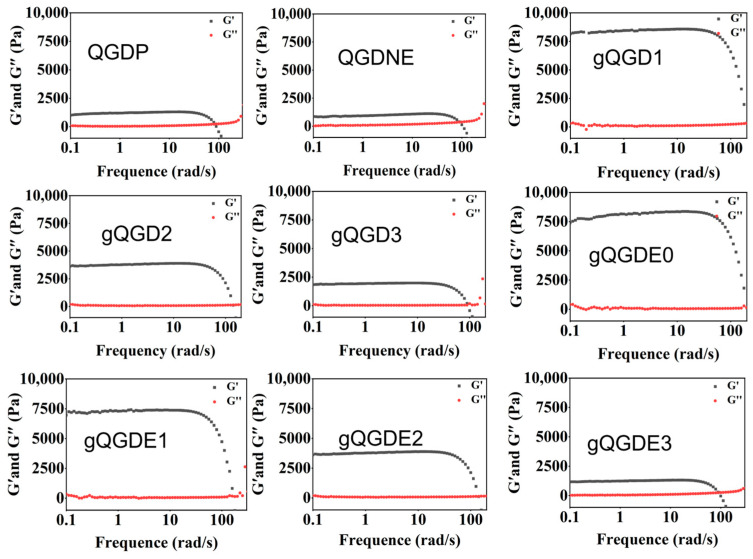
The storage modulus (G′) and loss modulus (G″) of the fabricated hydrogels were recorded in terms of frequency.

**Figure 6:**
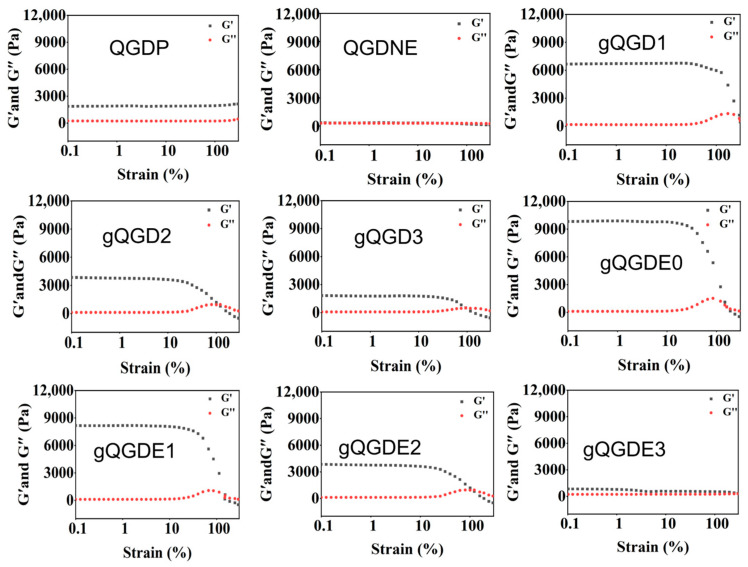
The storage modulus (G′) and loss modulus (G″) of the fabricated hydrogels were recorded in terms of strain.

**Figure 7:**
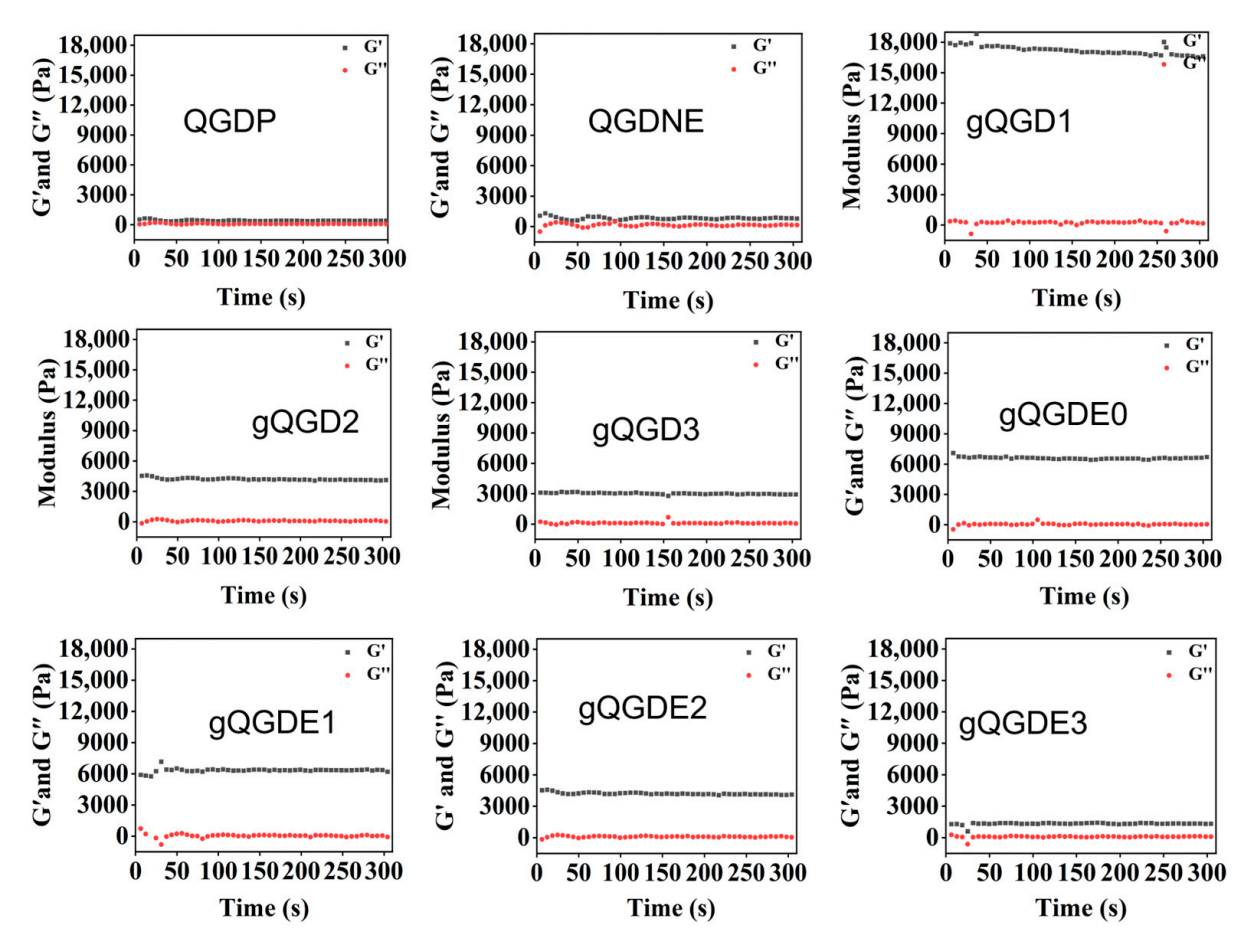
The storage modulus (G′) and loss modulus (G″) of the fabricated hydrogels were recorded in terms of time.

The rheological properties of all fabricated hydrogels were fully investigated using a rheometer. The storage modulus (G′) reflected the elasticity of the material, while the loss modulus (G″) characterized viscosity. The values of G′ and G″ for all hydrogels were recorded in terms of frequency, strain, and time. As shown in Figure 5, during the frequency sweep test, the G′ values of all groups of hydrogels were higher than G″, indicating they were stable viscoelastic solids with excellent elasticity. Among them, the hydrogels cross-linked by two cross-linkers, genipin and EDC/NHS, have a higher G′ than the gels cross-linked by only one of the cross-linkers, genipin or EDC/NHS. The G′ values of gQGD and gQGDE hydrogels were approximately 8000 Pa. This is because hydrogels crosslinked through the two crosslinking agents, which enhanced crosslinking density to increase the mechanical strength and stability. Furthermore, the storage modulus of hydrogels decreased along with the increase of EGCG in the hydrogels, demonstrating that the addition of EGCG might have a hindering effect on the crosslink density of hydrogels. The similar results were also obtained in strain weep and time sweep (Figure 6 and Figure 7). With the increase in strain, the G′ of sample QGDNE hydrogel decreased earlier compared to that of QGDP and gQGD hydrogels, confirming that the network structure could be easily destroyed. With further increase in strain, the crosspoint between G′ and G″ occurred, indicating the complete destruction of the hydrogel structure. Furthermore, all fabricated hydrogels exhibited stable G′ and G″ over time. These results showed that the prepared hydrogels possessed adjusted mechanical properties.

**Figure 8:**
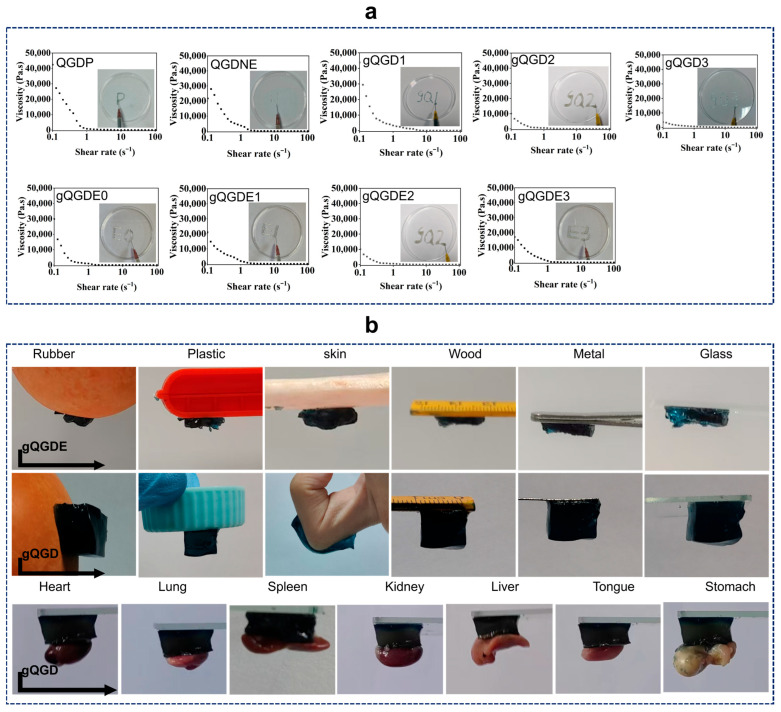
(**a**) The viscosities of the fabricated hydrogels over the shear rate, confirming their good injectability. The inset images illustrate that the hydrogels can be injected using a syringe. (**b**) The fabricated gQGDE and gQGD hydrogels exhibit good adhesive performance; the hydrogels could firmly adhere to the surface of different matrices, including rubber, plastic, ceramics, wood, metal, glass, skin, and rubber gloves and mouse organs of heart, lung, spleen, kidney, liver, tongue, and stomach.

In addition, the viscosities of all fabricated hydrogels as a function of shear rate were measured (Figure 8a). All hydrogels exhibited significant shear-thinning behavior, with viscosity decreasing gradually with increasing shear rate, indicating their injectability. It was observed that the hydrogels with different crosslinking approaches demonstrated the similar viscosity-shear rate profiles, indicating that the crosslinking approach employed this work does not influence the viscosity and injectability. The hydrogels could be injected using a 2.5 mL syringe, and the characters such as “gQ1”, “gQ1”, and “E0” could be easily written on the culture dish, as shown in the inserted photos in Figure 8a. The injection process was smooth without any blockages, confirming the excellent injectability of the hydrogel. This property endowed the hydrogel with excellent plasticity, enabling it to fill irregular wounds precisely, and it has great potential for clinical application [45].

### 2.6. Adhesive Performance

The adhesive properties of all hydrogels were investigated by adhering them to different materials and various organs (Figure S3, Supporting Information). The gQGDE and gQGD hydrogels exhibited excellent and long-lasting adhesion to various materials, including glass, human skin, rubber, plastic, wood, and metal (Figure 8b). Moreover, the gQGD hydrogels also displayed good adhesion to different organs, such as hearts, livers, spleens, tongue, stomach, lungs and kidneys of mice (Figure 8b). The excellent adhesion performance of the fabricated hydrogels mainly due to the hydrogen bonds, electrostatic interactions, and coordination bonding. Genipin crosslinked QCS hydrogels had demonstrated strong adhesion, especially due to the grafted dopamine groups in gelatin, and the fabricated hydrogels with genpin crosslinked QCS network and Gel-DA polymer, and the adhesion strength was improved. The obtained data confirmed that the fabricated hydrogels can be used for long-term fixation of wound dressings, the construction of a hemostatic and antibacterial barrier, and the facilitation of wound healing.

### 2.7. Self-Healing Behavior

**Figure 9:**
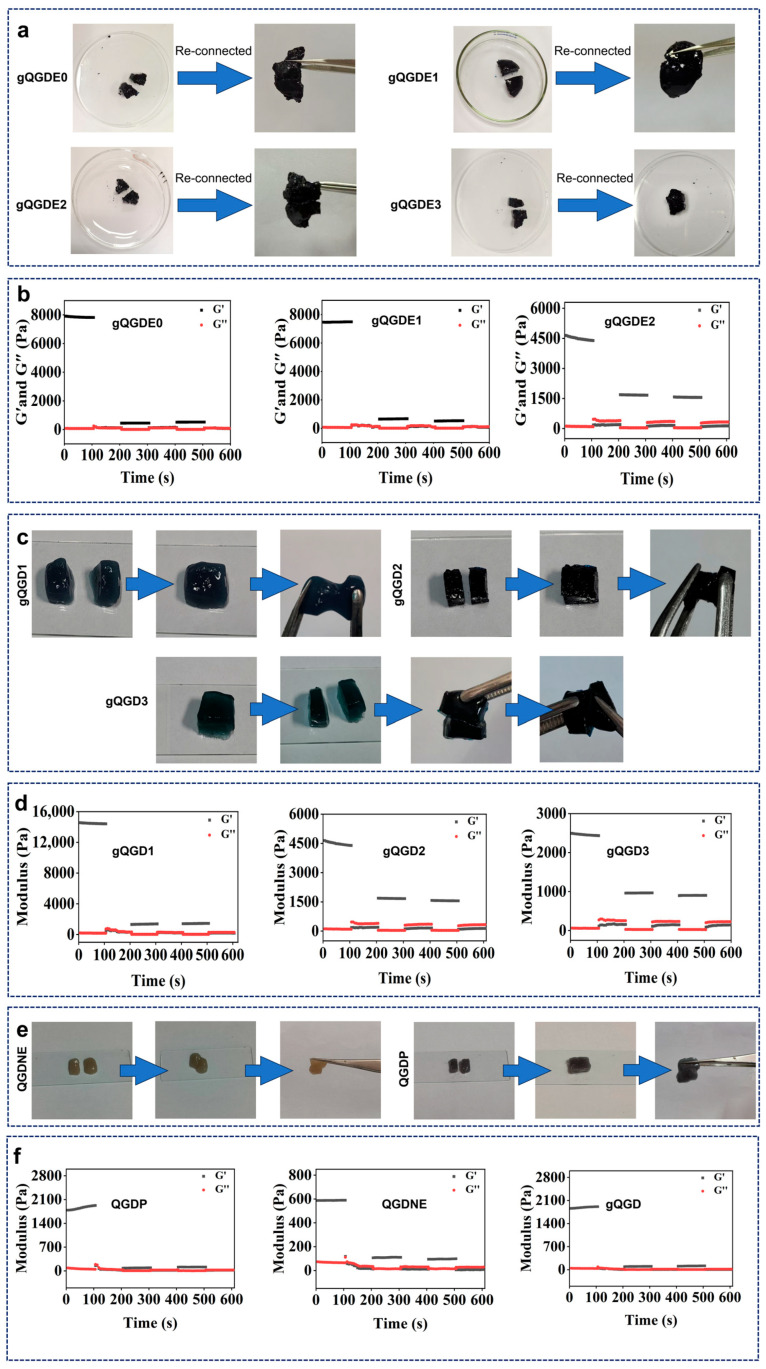
(**a**,**c**,**e**) Photos display that the fabricated hydrogels (gQGDE, gQGD, QGDP, and QGDNE) could be cut and reconnected, indicating their self-healing properties. (**b**,**d**,**f**) The self-healing behavior of the fabricated hydrogels was further confirmed using a 3-cycle step strain test (1% and 100%).

When a complete hydrogel of regular shape was cut and left to stand, and then pulled with forceps, the trace of the original cut disappeared, and it was not easily broken, as shown in Figure 9a,c,e, indicating that the hydrogel has self-healing ability. In the continuous step strain test (Figure 9b,d,f), at low strain, the structure of the hydrogel was damaged, and G′ of the hydrogel was greater than G″, indicating the stability of the hydrogel. At high strain (300%), the storage modulus G′ was lower than G″, indicating the collapse of the hydrogel network. Interestingly, when the strain was switched to low strain again, G′ was greater than G″, verifying its self-healing performance. The excellent self-healing abilities of the fabricated hydrogels are mainly due to the formation of Schiff base bonds after re-connecting, which was verified in FT-IR analysis (Figure 1f).

### 2.8. Antibacterial Properties

**Figure 10:**
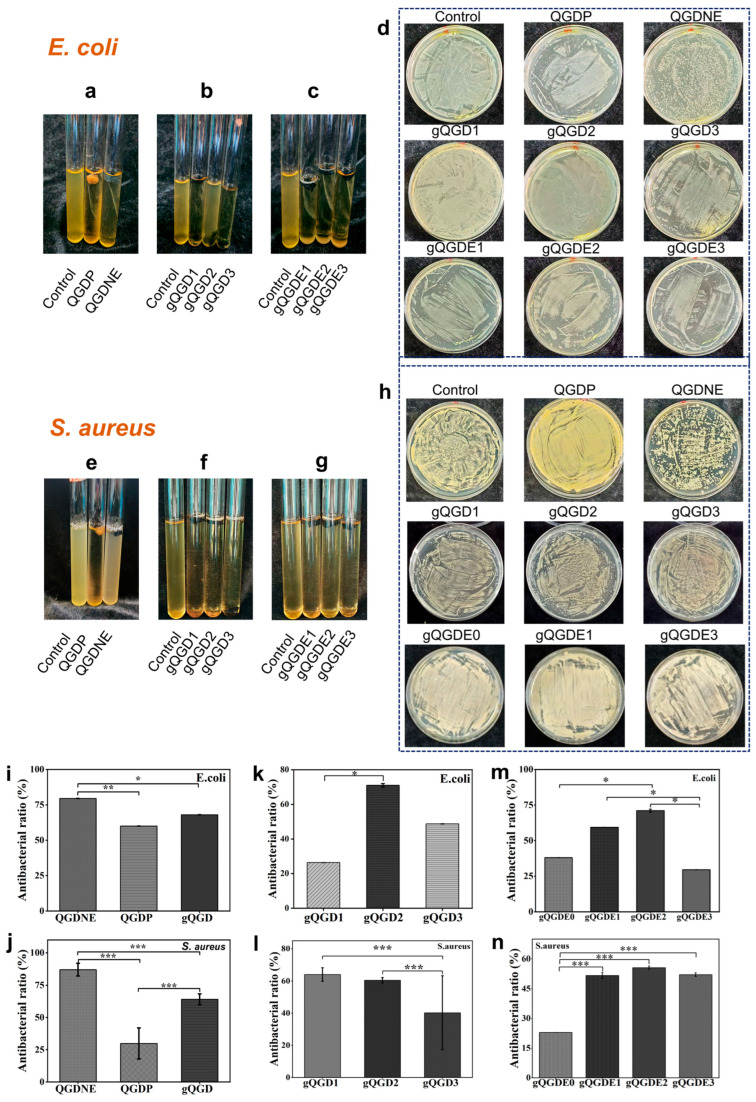
The antibacterial activity evaluation of the fabricated hydrogels. Photos exhibit the hydrogels in Luria Bertani (LB) agar liquid medium in a tube against (**a**–**c**) *E. coli* and (**e**–**g**) *S. aureus.* Photos display the hydrogels in a nutrient agar (NA) plate against (**d**) *E. coli* and (**h**) *S. aureus.* The antibacterial ratios of hydrogels against (**i**,**k**,**m**) *E. coli* and (**j**,**l**,**n**) *S. aureus.* * *p* < 0.05, ** *p* < 0.01, and *** *p* < 0.001.

Gram-positive bacteria (*S. aureus*) and Gram-negative bacteria (*E. coli*) were used to study the antibacterial activity of the hydrogel. The positively charged amino groups of QCS polymer electrostatically adsorb to the negatively charged surface of bacteria, disrupting the cell membrane [20]. Moreover, EGCG binds to the lipid bilayer of the cell membrane via negative charges, thereby interfering with membrane stability, inhibiting the activities of thioredoxin and reductase, blocking bacterial metabolism, and thereby suppressing proliferation [46]. The hydrogel demonstrated excellent antibacterial performance against both types of bacteria. Specifically, the hydrogel dressing with strong antibacterial properties can effectively prevent external bacteria from entering the wound, thereby reducing the risk of infection and promoting faster wound healing. The antibacterial properties of QGDNE hydrogels cross-linked by EDC and NHS were better than QGDP cross-linked by genipin, as well as that of gQGD cross-linked by genipin, EDC and NHS (Figure 10). Compared to other two groups, the antibacterial performance of gQGD2 was the best and the antibacterial rate of gQGD2 hydrogel against *S. aureus* and *E. coli* exceed 60% (Figure 10k,l), which was corresponded to antioxidative properties of gQGD2. In the gQGDE hydrogels with different amounts of EGCG, the gQGDE2 with 0.05 g of EGCG showed the best antibacterial performance, which was superior to gQGDE1 with 0.03 g and gQGDE3 with 0.07 g (Figure 10m,n).

### 2.9. Cytocompatibility and Hemocompatibility Evaluation

Cytocompatibility is a prerequisite for designing hydrogel dressings in wound healing applications. In the study, we cultured NIH-3T3 cells with hydrogel extracts and employed fluorescent microscopy, CCK-8 analysis, and cell scratch assays to investigate the cytocompatibility of the hydrogels.

**Figure 11:**
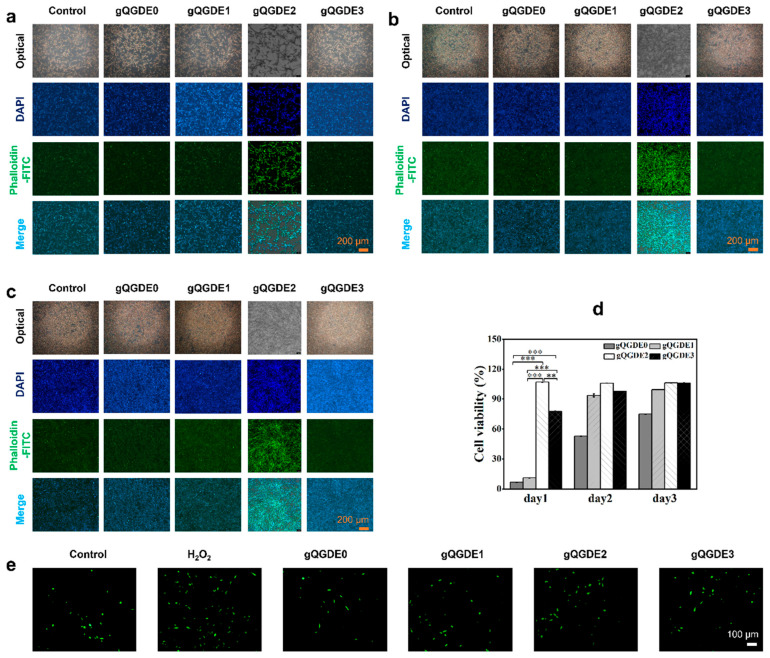
Optical photos and the fluorescent microscopy images at (**a**) day 1, (**b**) day 2, and (**c**) day 3 after culturing NIH 3T3 cells with the gQGDE hydrogels extracts, and the phalloidin-FITC/DAPI was used for staining. (**d**) Cell viability of NIH 3T3 cells after culturing with the gQGDE hydrogel extracts for days according to the CCK-8 assay, ** *p* < 0.01, *** *p* < 0.001. (**e**) The protective effect of the gQGDE hydrogels on intracellular ROS levels in NIH 3T3 cells, which were treated with H_2_O_2_, and DCFH-DA staining was used.

As shown in Figure 11a–c, cell numbers in all groups increased over time, indicating that NIH 3T3 cells could grow and proliferate in the gQGDE hydrogel extract. According to the CCK-8 assay, the absorbance of the control group increased from day 1 to day 3, indicating that the sample hydrogel extracts facilitated cell proliferation. All gQGDE hydrogel-treated groups showed increased cell viability over time (Figure 11d). These results indicate that hydrogels with EGCG at different time points exhibited better cell compatibility than gQGDE0 without EGCG, demonstrating the excellent biocompatibility of the hydrogels. In addition, the protective effect of hydrogels on NIH 3T3 cells was also assessed in response to the intracellular oxidative stress induced by H_2_O_2_. As seen in Figure 11e, compared to the control group, the H_2_O_2_-treated group exhibited strong green fluorescence, suggesting a large amount of ROS was produced in the cells. However, the hydrogel groups exhibited lower fluorescence intensity than other groups, indicating the hydrogels’ capacity to scavenge intracellular ROS. This is because EGCG can effectively eliminate various reactive oxygen species (ROS), including superoxide anion radicals, hydrogen peroxide, hydroxyl radicals, and singlet oxygen [47,48].

**Figure 12:**
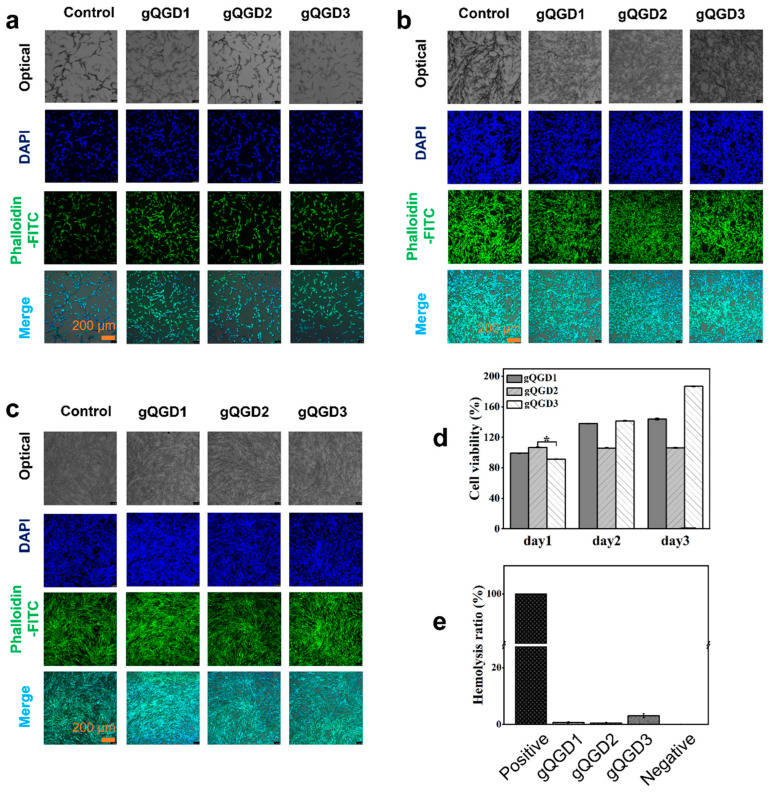
Optical photos and the fluorescent microscopy images of NIH 3T3 cells after culturing with the gQGD hydrogel extracts at (**a**) day 1, (**b**) day 2, and (**c**) day 3, and the phalloidin-FITC/DAPI was used for staining. (**d**) Cell viability of NIH 3T3 cells after culturing with the gQGD hydrogel extracts for 1, 2, and 3 days, measured by CCK-8 assay, * *p* < 0.05. (**e**) The hemolysis rates of the gQGD hydrogels.

In Figure 12a–d, the cell number in the gQGD hydrogel sample group increased steadily, with maintained morphology and no significant difference compared to the control group, further confirming its excellent cell compatibility. The hemolysis test results are shown in Figure 12e. The positive control group exhibited nearly 100% hemolysis, as in the Millipore water, the blood cell membranes broke, while the negative control group showed nearly 0% hemolysis, as 0.9% saline did not cause hemolysis, ensuring the reliability of the results. The hemolysis rates for the gQGD sample groups gQGD1, gQGD2, and gQGD3 were 0.75%, 0.61%, and 3.1%, respectively (all below 5%).

**Figure 13:**
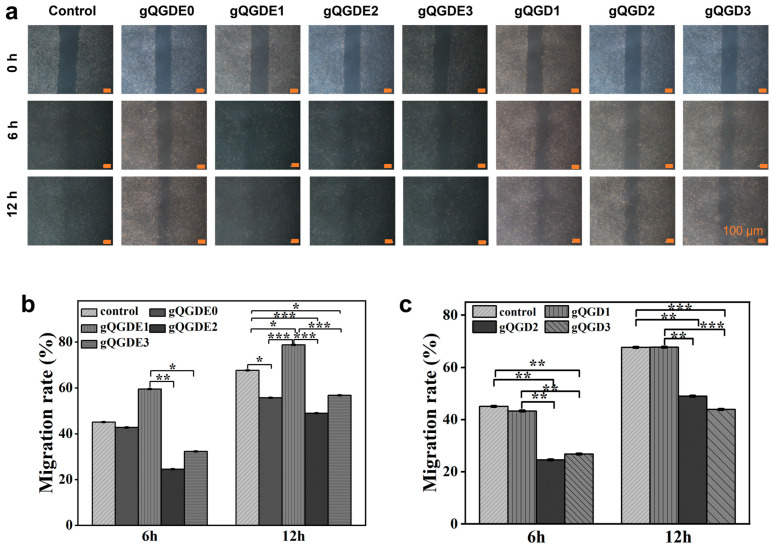
(**a**) Scratch assay using NIH 3T3 cells cultured with gQGDE and gQGD hydrogel extracts for 6 and 12 h. (**b**) Migration rates of NIH 3T3 cells of gQGDE. (**c**) Migration rates of NIH 3T3 cells of gQGD. * *p* < 0.05, ** *p* < 0.01, and *** *p* < 0.001.

Furthermore, the effects of hydrogels on NIH 3T3 cells were evaluated using a cell scratch assay. As shown in Figure 13, all hydrogels supported cell migration after 12 h of co-culture with hydrogel extracts. The cell migration rate in the gQGD hydrogel treatment group was similar to the control group (Control: 67.66%; gQGD1, gQGD2, gQGD3: 67.70%, 48.98%, 43.96%), indicating minimal impact on cell growth. The migration rates for gQGDE0, gQGDE1, gQGDE2, and gQGDE3 were 55.72%, 78.75%, 48.99%, and 56.77%, respectively, with the gQGDE0 control group at 67.66%.

## 3. Conclusions

In this paper, we have developed interpenetrated hydrogels based on DA-Gel, genipin-crosslinked QCS, and EGCG. The crosslinking approach and DA-Gel/QCS ratios can significantly regulate the main properties of hydrogels, including the interconnected microstructure, in vitro degradability, bioadhesive strength, mechanical properties, swelling ratios, and rheological behavior. The hydrogels possessed the expected self-healing ability and injectability. In particular, the hydrogels exhibited excellent adhesion to various materials and organs, including the heart, lung, spleen, kidney, liver, tongue, and stomach. Due to the inherent antibacterial properties of QCS and the incorporation of EGCG, the hydrogels exhibited strong antibacterial activity against *E. coli* and *S. aureus*, antioxidant activity, and ROS-scavenging activity. In addition to excellent cytocompatibility, hemocompatibility, and cell migration, the fabricated hydrogels have shown great potential for biomedical applications, such as bio-adhesives and wound-healing dressings.

## 4. Materials and Methods

### 4.1. Chemicals

Chitosan (CS, 100–200 mPa·s of viscosity, and Deacetylation Degree (DD) ≥ 95%) and N-Hydroxysuccinimide (NHS, 98%) were bought by Macklin Biochemical Co., Ltd. (Shanghai, China), gelatin (CP, viscosity ≥ 15 mm^2^/s) (Sinopharm Chemical Reagent Co., Ltd., Shanghai, China), genipin (98%) was gotten from Shanghai Shean chemical Co., Ltd. (Shanghai, China), (-)-Epigallocatechin-3-gallate (EGCG, >99.13%) was purchased from Picasso Group Co., Ltd. (Hongkong, China), ethanol acid (AG, ≥99.5%), anhydrous acetic acid (AR, 99.7%), 1-(3-dimethylaminopropyl)-3-ethylcarbodiimide hydrochloride (EDC, 98%) and N,N-Dimethylformamide (DMF, AR, 99.5%) were obtained from Ron Chemical Co., Ltd. (Shanghai, China). Dopamine hydrochloride (DA, 98%) and glycidyltrimethylammonium chloride (GTMAC, >95%) were purchased from Aladdlin (Shanghai, China), phosphate-buffered saline (PBS). Millipore water was produced by a Milli-Q 50 SP water purification system from the USA (Millipore Corporation, Billerica, MA). No further purification was performed on these reagents, and they were retained in their original condition from the supplier.

### 4.2. Preparation of Quaternized Chitosan (QCS) and Dopamine-Modified Gelatin (Gel-DA)

QCS was synthesized according to our previous report [19]. Briefly, 2 g of CS was dissolved in 72 mL of Millipore water in a baker. The CS solution was obtained after stirring at 450 rpm for around 30 min. After uniform dispersion, 1 mL of glacial acetic acid was added to the CS solution, and the mixture was stirred at 450 rpm for 30 min. Subsequently, 2 g of GTMAC was added to the above solution, and the mixture was stirred at 450 rpm for 15 h at 50 °C. The resulting solution was dialyzed in Millipore water using a dialysis bag (Molecular Weight Cut Off: 8000–14,000 kDa) for 5 days, with the Millipore water changed 3 times daily, followed by freeze-drying to obtain the QCS polymer material (flaky shape).

Dopamine was successfully grafted onto gelatin using EDC and NHS [39]. Firstly, 1 g of gelatin was dissolved in a mixed solution (18 mL) consisting of PBS and DMF, with a volume ratio of PBS/DMF of 1:1. The mixed solution was stirred (400 rpm) at 60 °C until completely dissolved. Meanwhile, 1.5 g of EDC and 0.9 g of NHS were added to 4 mL DMF, and the pH was adjusted to 5 for use. The obtained solution was stirred (400 rpm) for 30 min at ambient temperature until it turned limpid. Subsequently, 1 g DA was dissolved in 1 mL of Millipore water, and this solution was combined with the two solutions mentioned above and reacted for 24 h under N_2_ protection at 400 rpm. Finally, the mixture was dialyzed in Millipore water (MW 8000–14,000 kDa). The Millipore water was replaced three times a day, followed by lyophilization to obtain Gel-DA polymer (flaky shape).

### 4.3. Preparation of Hydrogels

First, based on QCS and Gel-DA, the hydrogels with different crosslinking approaches were fabricated. We chose a QCS: Gel-DA mass ratio of 7:3 to prepare hydrogels with different crosslinking agents and to investigate their effects on gelation and hydrogel properties. One hydrogel was prepared using only genipin, while another was prepared using only EDC/NHS. They were named QGDP and QGDNE, respectively, and the hydrogel crosslinked with both Genipin and EDC/NHS was named gQGDE (Table 1).

Secondly, we prepared hydrogels with varying mass ratios of the macromolecular substances QCS and Gel-DA. Briefly, different amount of QCS and Gel-DA were dissolved in Millipore water (20 mL, 4 mL), respectively. 1.5% genipin solution in ethanol and 2.5% EGCG solution were prepared using Millipore water. Then, 0.2094 g of EDC and 0.075 g of NHS were added to the prepared Gel-DA solution, then stirred in an ice-water bath for approximately 10 min [49]. After all of the Gel-DA solution was mixed with the total amount of the previous QCS solution, the prepared genipin and EGCG solution was added. By varying the mass ratios of the materials, hydrogels with different compositions were prepared and designated as gQGD1, gQGD2, and gQGD3 (Table 2).

To investigate the effects of varying EGCG concentration on the properties of the hydrogel, a QCS/Gel-DA ratio of 6:4 was used to prepare hydrogels with different EGCG concentrations, which were named gQGDE (Table 3).

### 4.4. Fourier-Transform Infrared Spectroscopy (FT-IR) Analysis

To verify that the preparation of the materials and fabrication of the hydrogels was successful, Fourier-Transform Infrared Spectroscopy (FT-IR) analysis was conducted. Briefly, materials and hydrogels were analyzed using FT-IR (Bruker, Bremen, Germany, VERTEX70) in attenuated total reflectance (ATR) mode. FT-IR spectra were obtained in the wavenumber range of 500–4000 cm^−1^ with a resolution of 1 cm^−1^, and 64 scans for all spectra.

### 4.5. H Nuclear Magnetic Resonance (^1^H NMR) Spectroscopy

For confirming the successful synthesis of QCS and Gel-DA, the NMR spectrometer at 600 MHz (JEOL ECZ600R/S3, JEOL, Tokyo, Japan) was employed. The grafting degrees were also measured according to ^1^H NMR spectra. 20 mg of polymers (QCS, CS, Gel-DA, Gelatin, Dopamine) were dissolved in 500 μL of D_2_O and then transferred to a standard NMR tube for ^1^H NMR characterization through an NMR spectrometer at 600 MHz (JEOL ECZ600R/S3, JEOL, Tokyo, Japan).

### 4.6. Scanning Electron Microscopy (SEM) Testing

To more intuitively observe the porous structure of the hydrogel, the SEM test was conducted. In brief, the freeze-dried hydrogel was cut into thin slices. Before observation with a cold-field emission scanning electron microscope (SEM; Chiyoda-ku, Tokyo, Japan; Hitachi S-4800), a platinum layer was sputtered onto the sample surface for 40 s; thereafter, the microtopography and pore structure of the hydrogel were characterized using SEM. Pore sizes were measured using ImageJ software (Version 1.53).

### 4.7. Rheological Property Testing

The rheological properties of the hydrogels were tested at 37 °C using a TA rheometer (DHR-2, TA Instruments Co., Ltd., New Castle, DE, USA) with a constant strain of 1% and a constant frequency of 10 rad/s. The storage modulus (G′) and loss modulus (G”) were measured using a 20 mm diameter aluminum platform [50]. The fixture edges were sealed with silicone oil to prevent water loss from the hydrogel. Subsequently, the linear viscoelastic region of the hydrogel was determined by undertaking an oscillating strain sweep experiment. The angular frequency was maintained at *ω* = 10 rad s^−1^. After the dynamic strain scanning tests were conducted, the strain ranged from 0.1% to 500%. The frequency sweep test was carried out by keeping the hydrogel spline at a constant condition of γ = 0.5%, and the frequency range was applied from 0.01 rad s^−1^ to 500 rad s^−1^ [20].

### 4.8. Injectability Test

The injectable performance of the hydrogels was measured at 25 °C using a TA rheometer with a constant strain of 1%, while the shear rate was varied from 0.1 rad/s to 100 rad/s. Twenty oscillatory frequency sweep experiments were performed [20]. Moreover, the prepared hydrogel was loaded into a syringe and extruded to write the characters “gQ1”, etc.

### 4.9. Adhesive Strength Test of Hydrogels

The adhesive capability of the hydrogels was assessed by contacting them with various materials, such as mouse heart, liver, spleen, lung, kidney, glass, wood, and rubber, etc., which were documented in photographs, showcasing the hydrogels’ effectiveness in adhering to different materials. The self-strength of hydrogels was quantitatively studied using a lap-shear test. In the test, the porcine skin was first cut into rectangles of about 40 mm × 25 mm. Then, the rectangular hydrogels with a size of 20 mm × 15 mm was placed at the end of the skin [18]. Another skin was bonded with the other side of hydrogel to form spliced porcine skins and placed for 10 min at room temperature. Finally, the adhesive strength of hydrogels was evaluated on a universal testing apparatus (CMT-4103, Zhuhai Junjie Technology Co., Ltd., Zhuhai, China).

### 4.10. Self-Healing Performance Testing

To evaluate the self-healing performance, the hydrogel was placed between the parallel plates of a TA rheometer through a continuous step strain time sweep, in which the amplitude oscillatory strain was alternated between γ = 1.0% and γ = 100% for three cycles, with each strain interval lasting 100 s [20]. Furthermore, the macroscopic self-healing performance was tested. The hydrogels were cut into two parts. The two parts were contacted for 1 h to accomplish the process at 37 °C. The results were recorded through photography.

### 4.11. Equilibrium Swelling Performance Testing

The equilibrium swelling ratios of the hydrogels were measured in PBS (pH 7.4) at 37 °C. At predetermined time points, the hydrogels were removed from PBS, and the surface water was gently removed with filter paper. The weight of the water-absorbed hydrogel was kept in an equilibrium state. Then, the hydrogel was split into four equal portions, with each portion weighed individually. The weight was recorded as *W_s_*. The hydrogels were freeze-dried and weighed, and the weight was recorded as *Wₐ*. The swelling ratio of hydrogels was calculated through the following formula [51]:

$$\mathrm{Swelling}\;\mathrm{Ratio}\;(\mathrm{SR})\;(\%)\;=\frac{W_{s}-W_{a}}{W_{a}}\times 100\%$$

### 4.12. Mechanical Property of Hydrogels

To assess the mechanical properties of the hydrogel, we conducted the compression test [19]. The compressive strength of the cylindrical hydrogel (6 mm in diameter and 7.5 mm in height) was measured using mechanical testing equipment (CMT-4103, Zhuhai Junjie Tech. Co., Ltd., Zhuhai, China) at a probe speed of 1 mm/min.

### 4.13. Antioxidant Performance Testing

The 1, 1-diphenyl-2-picrylhydrazyl (DPPH) free-radical scavenging assay was used to investigate the antioxidant activity of the hydrogels [32]. Briefly, 4 mg of hydrogel was dissolved in 5 mL of 0.1 mmol/L DPPH solution to evaluate its DPPH radical scavenging ability. The mixture was placed in a dark tube and incubated at 25 °C for 30 min with shaking at 150 rpm. The absorbance of the supernatant at 517 nm was measured using a microplate reader (Synergy H1, BioTek, Shanghai, China). The DPPH scavenging rate of hydrogels was calculated using the formula:

$$\mathrm{DPPH}\;\mathrm{Scavenging}\;\mathrm{Rate}\;(\%)\;=\frac{A_{0}\;-\;A_{1}}{A_{0}}\times 100\%$$

where *A*_0_ represents the absorbance of the control group (without hydrogel), and *A*_1_ represents the absorbance of the sample group.

### 4.14. Antibacterial Performance Testing

*S. aureus* (ATCC 6538) and *E. coli* (ATCC 25922) were used to evaluate the antibacterial properties of the hydrogel according to the previous report [52]. Briefly, 0.015 g of hydrogel was added to 10 mL of LB medium containing 10 μL of bacterial suspension of *E. coli* (1 × 10^7^ CFU mL^−1^) or *S. aureus* (1 × 10^7^ CFU mL^−1^). The tube containing 10 mL of LB medium with 10 μL of bacterial suspension without hydrogel was used as the control group. Then the mixed solution was cultured at 37 °C for 8 h at 180 rpm. Finally, the absorbance at 600 nm (OD_600_) was measured using a UV-Vis spectrophotometer. Three replicates were performed for each sample group. The antibacterial rate was calculated as follows:

$$Antibacterial\;Rate\;(\%)=\frac{{OD}_{control}-{OD}_{sample}}{{OD}_{control}}\times 100\%$$

where *OD_control_* and *OD_sample_* are the absorption values of the blank control group and the sample group, respectively.

### 4.15. Cytocompatibility of Hydrogels

To evaluate the cytocompatibility of the hydrogel, we conducted the Cell Counting Kit-8 (CCK-8) test to assess the hydrogel’s toxicity to mouse embryonic fibroblast cells (NIH 3T3 cells), obtained from ATCC (CRL-1658™, Manassas, WV, USA) [52]. Before conducting the experiment, we first prepared the hydrogel extract solution. In summary, the tested samples were pre-treated by immersing in 75 *v*/*v*% alcohol and irradiation with ultraviolet light for 12 h, then thoroughly soaked and washed with sterile PBS more than 3 times to completely remove the ethanol. Finally, add an equal amount of complete medium to each sample (prepared according to the Dulbecco’s modified Eagle medium (DMEM) ratio: add 10 *v*/*v*% fetal bovine serum (FBS) and 1 *v*/*v*% penicillin-streptomycin, according to ATCC instructions), incubate at 37 °C for 24 h, and prepare the hydrogel extract solution.

CCK-8 test: the NIH 3T3 cells in the logarithmic growth phase were used to prepare a cell suspension, which was added to a 96-well plate (100 μL per well) with around 5000 cells in each well. Subsequently, the NIH 3T3 cells were co-cultured with the hydrogel extract solution and cultured under a 5% CO_2_ humidified incubator at 37 °C for 1, 2, and 3 days, respectively. The experiment was divided into a blank group (complete culture medium only) and gQGD hydrogel sample groups. Then, at each expected experimental time, 10% (by culture volume) of CCK-8 solution was added to each well and co-incubated at 37 °C for 2 h. Finally, the absorbance (A) at 450 nm was measured using a microplate reader to assess cell viability.

Phalloidin-FITC/DAPI Staining: The cytocompatibility of the hydrogel was investigated by co-culturing NIH 3T3 cells with hydrogel extracts. After co-culturing NIH 3T3 cells with hydrogel extract for 1, 2, and 3 days, cell nuclei and cytoskeletons were double-stained with DAPI/phalloidin reagents, respectively. Then, cell morphology was observed under an inverted fluorescence microscope.

Cell Migration Assay: The cell migration experiment was accomplished by using NIH 3T3 cells containing hydrogel extract in a 6-well plate to reflect the activity of the NIH 3T3 cells [53]. The cell-scratch plug-in device (ibidi culture-insert 2 well, ibidi GmbH, Martinsried, Germany) was used to cultivate NIH 3T3 cells, with 50,000 cells seeded in each scratch plug well. The growth of cells was observed and recorded under an optical microscope at 0 h, 6 h and 12 h, respectively. Through the ImageJ software (Version 1.53), the migration rate can be statistically calculated based on the following equation:

$$Migration\;Rate\;(\%)\;=\frac{A_{0}-A_{t}}{A_{0}}\times 100\%$$

where *A*_0_ indicates the blank area of the scratch at 0 h, and *A_t_* represents the blank area of the scratch at 6 h or 12 h.

### 4.16. In Vitro Hemolysis Test

The hemolytic properties of the hydrogel were tested to determine whether it would cause red blood cell rupture and, consequently, lead to hemolysis, as reported in the previous literature [54]. Fresh blood collected from Sprague-Dawley (SD) rats was stored in EDTA-anticoagulated tubes and centrifuged at 3000 rpm for 10 min. Then, the red blood cells (RBCs) were washed with 0.9% saline. This process was repeated several times until the supernatant was clear. Subsequently, 2.5 mg of the hydrogel sample was added to a centrifuge tube containing 1 mL of 0.9% saline (*A_s_*) and incubated at 37 °C for 30 min. Then, 20 μL of the RBCs suspension was added to the tube and incubated at 37 °C for 1 h. The absorbance at 540 nm was measured using a microplate reader (PerkinElmer, Shelton, USA). The positive control (*A_p_*) consisted of 1 mL of ultrapure water mixed with 20 μL of RBC suspension. The negative control (*A_n_*) consisted of 1 mL of 0.9% saline mixed with 20 μL of RBC suspension. The hemolysis rate was calculated as follows:

$$\mathrm{Hemolysis}\;\mathrm{Rate}\;(\%)\;=\frac{A_{s}-A_{n}}{A_{p}-A_{n}}\times 100\%$$

### 4.17. In Vitro Degradation Test

To verify the stability of the hydrogels, subject the hydrogels to in vitro degradation experiments. In brief, 0.01 g of dried hydrogel sample was weighed and recorded as the initial mass (*M*_0_, g). The weighed sample was immersed in 5 mL of PBS and placed in a shaker at 37 °C with continuous shaking at 50 rpm. At predetermined time intervals, samples were removed, freeze-dried again, and weighed to determine the remaining mass (*M_t_*, g). The biodegradation rate was calculated using the following formula:

$$\mathrm{Degradation}\;\mathrm{Rate}\;(\%)\;=\frac{M_{0}-M_{t}}{M_{0}}\times 100\%$$

where *M*_0_ and *M_t_* represent the initial freeze-dried mass of the hydrogel and the freeze-dried mass at specified time points after immersion in PBS, respectively.

### 4.18. In Vitro EGCG Release Experiment

The in vitro EGCG release experiment was carried out to demonstrate the hydrogel’s EGCG release capacity [55]. Briefly, Freeze-dried composite hydrogels with different EGCG contents (0.03 g) were immersed in 50 mL of phosphate-buffered saline (PBS, pH = 7.2) at 37 °C and placed in a shaker at 50 rpm. At predetermined time intervals, 3 mL of the supernatant was withdrawn, and its absorbance at 273 nm was measured using a UV-Vis spectrophotometer (Lambda 950, Waltham, MA, USA). Simultaneously, 3 mL of fresh PBS was added back to the PBS buffer to maintain a constant volume. The time of the EGCG release experiment was 6 h in total. The concentration of EGCG released from the composite hydrogels was analyzed using a standard curve of EGCG.

### 4.19. Statistical Analysis

The experimental results were presented in the form of mean ± standard deviation (SD). Univariate or two-factor repeated measures ANOVA and graph basis tests were conducted using SPSS software (SPSS Inc., Chicago, IL, USA, Version 26.0.0.0) with statistical significance defined according to *p*-values of <0.05, <0.01 and <0.001, which represent 95%, 99%, and 99.9% confidence, respectively.

## Figures and Tables

**Table 1:** The composition of QCS, Gel-DA, genipin, and EDC/NHS is used to fabricate different hydrogels.

Sample	QCS	Gel-DA	Genipin	EDC/NHS
gQGD	0.7 g	0.3 g	0.03 g	0.2018/0.0748 g
QGDP	0.7 g	0.3 g	0.03 g	0 g
QGDNE	0.7 g	0.3 g	0 g	0.2018/0.0748 g

**Table 2:** Feed ratios for different gQGD hydrogel samples.

Sample	QCS	Gel-DA	Genipin	EGCG
gQGD1	0.7 g	0.3 g	0.03 g	0.05 g
gQGD2	0.6 g	0.4 g	0.03 g	0.05 g
gQGD3	0.5 g	0.5 g	0.03 g	0.05 g

**Table 3:** Feed ratios for hydrogels with different EGCG content.

Sample	QCS	Gel-DA	Genipin	EGCG
gQGDE0	0.6 g	0.4 g	0.03 g	0 g
gQGDE1	0.6 g	0.4 g	0.03 g	0.03 g
gQGDE2	0.6 g	0.4 g	0.03 g	0.05 g
gQGDE3	0.6 g	0.4 g	0.03 g	0.07 g

## Data Availability

Data will be made available on request of the authors.

## Supplementary Materials

The following supporting information can be downloaded at: https://www.mdpi.com/article/10.3390/gels12010067/s1, Figure S1: (a) Stress–strain curves of QGDP, QGDNE, and gQGD hydrogels during the tensile strength test. (b) Stress–strain curves of the gQGD hydrogels; Figure S2: (a) ABTS scavenging rates of the gQGD hydrogels, * *p* < 0.05. (b) Photos recorded display the hydrogels immersed in ABTS solution during the ABTS scavenging test; Figure S3: (a) The fabricated gQGD hydrogels could firmly adhere to different matrix surfaces, including rubber, plastic, ceramics, wood, metal, glass, skin, and rubber gloves. (b) The fabricated gQGDE hydrogels exhibit good adhesive performance, the hydrogels could firmly adhere to the surface of different matrix, including rubber, plastic, ceramics, wood, metal, glass, and pig skin. (c) The gQGD hydrogels exhibit good adhesive performance on different organs, including heart, lung, spleen, kidney, liver, tongue, and stomach.

## References

[1] Patrickios C.S. (2010). Polymer networks: Recent developments. Macromolecular Symposia.

[2] Chen R., Yang S., Liu B., Liao Y. (2023). Eco-friendly semi-interpenetrating polymer network hydrogels of sodium carboxymethyl cellulose/gelatin for methylene blue removal. Materials.

[3] Jia C., Zhang X., Li Y., Jiang Y., Zhang M., Lu P., Chen H. (2018). Synthesis and characterization of bio-based PA/EP interpenetrating network polymer as coating material for controlled release fertilizers. J. Appl. Polym. Sci..

[4] Sánchez-Cid P., Jiménez-Rosado M., Romero A., Pérez-Puyana V. (2022). Novel trends in hydrogel development for biomedical applications: A review. Polymers.

[5] Alinezhad V., Ghodsi R., Bagheri H., Beram F.M., Zeighami H., Kalantari-Hesari A., Salarilak L., Mostafavi E., Ahmadian Z., Shahbazi M.-A. (2024). Antioxidant, hemostatic, and injectable hydrogels with photothermal antibacterial activity to accelerate full-thickness wound regeneration. New J. Chem..

[6] Zhao N., Yuan W. (2023). Self-healing and shape-adaptive nanocomposite hydrogels with anti-inflammatory, antioxidant, antibacterial activities and hemostasis for real-time visual regeneration of diabetic wounds. Compos. Part B Eng..

[7] Zhong Y., Seidi F., Wang Y., Zheng L., Jin Y., Xiao H. (2022). Injectable chitosan hydrogels tailored with antibacterial and antioxidant dual functions for regenerative wound healing. Carbohydr. Polym..

[8] Yang D. (2022). Recent advances in hydrogels. Chem. Mater..

[9] Huang Y., Jayathilaka P.B., Islam M.S., Tanaka C.B., Silberstein M.N., Kilian K.A., Kruzic J.J. (2022). Structural aspects controlling the mechanical and biological properties of tough, double network hydrogels. Acta Biomater..

[10] Ning X., Huang J., A Y., Yuan N., Chen C., Lin D. (2022). Research advances in mechanical properties and applications of dual network hydrogels. Int. J. Mol. Sci..

[11] Farooq U., Teuwen J., Dransfeld C. (2020). Toughening of Epoxy Systems with Interpenetrating Polymer Network (IPN): A Review. Polymers.

[12] Yin Y., Gu Q., Liu X., Liu F., McClements D.J. (2023). Double network hydrogels: Design, fabrication, and application in biomedicines and foods. Adv. Colloid Interface Sci..

[13] Bhardwaj V., Harit G., Kumar S. (2012). Interpenetrating polymer network (IPN): Novel approach in drug delivery. Int. J. Drug Dev. Res..

[14] Wahid F., Hu X.-H., Chu L.-Q., Jia S.-R., Xie Y.-Y., Zhong C. (2019). Development of bacterial cellulose/chitosan based semi-interpenetrating hydrogels with improved mechanical and antibacterial properties. Int. J. Biol. Macromol..

[15] Matica M.A., Aachmann F.L., Tøndervik A., Sletta H., Ostafe V. (2019). Chitosan as a Wound Dressing Starting Material: Antimicrobial Properties and Mode of Action. Int. J. Mol. Sci..

[16] Shariatinia Z. (2018). Carboxymethyl chitosan: Properties and biomedical applications. Int. J. Biol. Macromol..

[17] Qu J., Zhao X., Liang Y., Zhang T., Ma P.X., Guo B. (2018). Antibacterial adhesive injectable hydrogels with rapid self-healing, extensibility and compressibility as wound dressing for joints skin wound healing. Biomaterials.

[18] Nie L., Wang L., Hu S., Wei Z., Ding X., Lu Y., Tang H., Ding P. (2025). Dopamine-conjugated hyaluronic acid hydrogel interpenetrated by genipin crosslinked quaternary ammonium chitosan for potential biomedical adhesives applications. Colloids Surf. B Biointerfaces.

[19] Wang L., Ding X., Li J., Li M., Ding P., Guo W., Wu Q., Sun Y., Jiang G., Okoro O.V. (2024). Genipin crosslinked quaternary ammonium chitosan hydrogels for wound dressings. Biomed. Mater..

[20] Wang L., Ding X., He X., Tian N., Ding P., Guo W., Okoro O.V., Sun Y., Jiang G., Liu Z. (2024). Fabrication and Properties of Hydrogel Dressings Based on Genipin Crosslinked Chondroitin Sulfate and Chitosan. Polymers.

[21] Ulubayram K., Aksu E., Gurhan S.I.D., Serbetci K., Hasirci N. (2002). Cytotoxicity evaluation of gelatin sponges prepared with different cross-linking agents. J. Biomater. Sci. Polym. Ed..

[22] Wang T.-W., Sun J.-S., Wu H.-C., Tsuang Y.-H., Wang W.-H., Lin F.-H. (2006). The effect of gelatin–chondroitin sulfate–hyaluronic acid skin substitute on wound healing in SCID mice. Biomaterials.

[23] Zaupa A., Neffe A.T., Pierce B.F., Nöchel U., Lendlein A. (2011). Influence of tyrosine-derived moieties and drying conditions on the formation of helices in gelatin. Biomacromolecules.

[24] Pourshahrestani S., Zeimaran E., Kadri N.A., Mutlu N., Boccaccini A.R. (2020). Polymeric Hydrogel Systems as Emerging Biomaterial Platforms to Enable Hemostasis and Wound Healing. Adv. Healthc. Mater..

[25] Guo J., Wang W., Hu J., Xie D., Gerhard E., Nisic M., Shan D., Qian G., Zheng S., Yang J. (2016). Synthesis and characterization of anti-bacterial and anti-fungal citrate-based mussel-inspired bioadhesives. Biomaterials.

[26] Gowda A.H.J., Bu Y., Kudina O., Krishna K.V., Bohara R.A., Eglin D., Pandit A. (2020). Design of tunable gelatin-dopamine based bioadhesives. Int. J. Biol. Macromol..

[27] Huang A., Honda Y., Li P., Tanaka T., Baba S. (2019). Integration of Epigallocatechin Gallate in Gelatin Sponges Attenuates Matrix Metalloproteinase-Dependent Degradation and Increases Bone Formation. Int. J. Mol. Sci..

[28] Rose J.B., Pacelli S., Haj A.J.E., Dua H.S., Hopkinson A., White L.J., Rose F.R.A.J. (2014). Gelatin-Based Materials in Ocular Tissue Engineering. Materials.

[29] Tsai C.C., Huang R.N., Sung H.W., Liang H.C. (2000). In vitro evaluation of the genotoxicity of a naturally occurring crosslinking agent (genipin) for biologic tissue fixation. J. Biomed. Mater. Res..

[30] Hu J., Webster D., Cao J., Shao A. (2018). The safety of green tea and green tea extract consumption in adults–results of a systematic review. Regul. Toxicol. Pharmacol..

[31] Kwon Y.-S., Kim H.-J., Hwang Y.-C., Rosa V., Yu M.-K., Min K.-S. (2017). Effects of epigallocatechin gallate, an antibacterial cross-linking agent, on proliferation and differentiation of human dental pulp cells cultured in collagen scaffolds. J. Endod..

[32] Nie L., Wei Q., Sun M., Ding P., Wang L., Sun Y., Ding X., Okoro O.V., Jiang G., Shavandi A. (2023). Injectable, self-healing, transparent, and antibacterial hydrogels based on chitosan and dextran for wound dressings. Int. J. Biol. Macromol..

[33] Lu W., Rahimnejad M., Sahadi B.O., Bottino M.C. (2025). Dopamine-Conjugated Methacrylated Gelatin Hydrogel—Physical, Mechanical, and Biological Properties. Gels.

[34] Zhou D., Li S., Pei M., Yang H., Gu S., Tao Y., Ye D., Zhou Y., Xu W., Xiao P. (2020). Dopamine-modified hyaluronic acid hydrogel adhesives with fast-forming and high tissue adhesion. ACS Appl. Mater. Interfaces.

[35] Maitra J., Shukla V.K. (2014). Cross-linking in hydrogels—A review. Am. J. Polym. Sci..

[36] Hu W., Wang Z., Xiao Y., Zhang S., Wang J. (2019). Advances in crosslinking strategies of biomedical hydrogels. Biomater. Sci..

[37] Fu J., Yap J.X., Leo C., Chang C.K., Show P.-L. (2024). Polysaccharide hydrogels for controlling the nutrient release. Sep. Purif. Rev..

[38] Foudazi R., Zowada R., Manas-Zloczower I., Feke D.L. (2023). Porous hydrogels: Present challenges and future opportunities. Langmuir.

[39] Martinez A.W., Caves J.M., Ravi S., Li W., Chaikof E.L. (2014). Effects of crosslinking on the mechanical properties, drug release and cytocompatibility of protein polymers. Acta Biomater..

[40] Zhang X., Li Y., He D., Ma Z., Liu K., Xue K., Li H. (2021). An effective strategy for preparing macroporous and self-healing bioactive hydrogels for cell delivery and wound healing. Chem. Eng. J..

[41] Zheng W., Yang W., Wei W., Liu Z., Tremblay P.L., Zhang T. (2024). An Electroconductive and Antibacterial Adhesive Nanocomposite Hydrogel for High-Performance Skin Wound Healing. Adv. Healthc. Mater..

[42] Feng W., Wang Z. (2023). Tailoring the swelling-shrinkable behavior of hydrogels for biomedical applications. Adv. Sci..

[43] Kamata H., Li X., Chung U.-i., Sakai T. (2015). Design of hydrogels for biomedical applications. Adv. Healthc. Mater..

[44] Nie L., Li X., Zhang T., Lu Y., Ding P. (2025). Schiff base formed functional hydrogel dressing via ϵ-poly-L-lysine modified chitosan and oxidized dextran with the incorporation of epigallocatechin-3-gallate. Biomed. Mater..

[45] Fang W., Yang L., Chen Y., Hu Q. (2023). Bioinspired multifunctional injectable hydrogel for hemostasis and infected wound management. Acta Biomater..

[46] Reygaert W.C. (2014). The antimicrobial possibilities of green tea. Front. Microbiol..

[47] Yuan M., Hu L., Zhu C., Li Q., Tie H., Ruan H., Wu T., Zhang H., Xu L. (2025). Comparison and Assessment of Anti-Inflammatory and Antioxidant Capacity Between EGCG and Phosphatidylcholine-Encapsulated EGCG. J. Cosmet. Dermatol..

[48] Su Z., Hu Y., Meng L., Ouyang Z., Li W., Zhu F., Xie B., Wu Q. (2024). Mussel-inspired Methacrylic Gelatin-dopamine/Ag Nanoparticles/Graphene Oxide Hydrogels with Improved Adhesive and Antibacterial Properties for Applications as Wound Dressings. J. Wuhan Univ. Technol.-Mater. Sci. Ed..

[49] Yan L., Liu S., Wang J., Ding X., Zhao Y., Gao N., Xia Z., Li M., Wei Q., Okoro O.V. (2024). Constructing Nerve Guidance Conduit using dECM-Doped Conductive Hydrogel to Promote Peripheral Nerve Regeneration. Adv. Funct. Mater..

[50] Zhou J., Wang Z., Yang C., Zhang H., Fareed M.S., He Y., Su J., Wang P., Shen Z., Yan W. (2022). A carrier-free, dual-functional hydrogel constructed of antimicrobial peptide Jelleine-1 and 8Br-cAMP for MRSA infected diabetic wound healing. Acta Biomater..

[51] Budianto E., Amalia A. (2020). Swelling behavior and mechanical properties of Chitosan-Poly(N-vinyl-pyrrolidone) hydrogels. J. Polym. Eng..

[52] Ding P., Ding X., Liu X., Lu Y., Zhao Y., Chu Y., Fan L., Nie L. (2024). Injectable, self-healing, antibacterial hydrogel dressing based on oxidized dextran and sialic acid substituted chitosan with incorporation of tannic acid. Eur. Polym. J..

[53] Nie L., Ding X., Lin Y., Yan L., Ding P., Yan L. (2024). Self-healing, adhesive, antibacterial, and biocompatible hydrogel dressings using sialic acid substituted chitosan and oxidized pullulan with incorporation of epigallocatechin gallate. Compos. Commun..

[54] Yoo S., Chang Y., Kim S., Yi G.-R., Hwang D.S. (2024). Unraveling the cellular mechanisms of tissue regeneration based on colloidal supraball bio-adhesives. Chem. Eng. J..

[55] Ding P., Sun Y., Jiang G., Nie L. (2025). Hydroxyproline-Modified Chitosan-Based Hydrogel Dressing Incorporated with Epigallocatechin-3-Gallate Promotes Wound Healing Through Immunomodulation. Gels.

