# Supplementary material for: Biomedical Interpenetrated Hydrogels Fabricated via Quaternary Ammonium Chitosan and Dopamine-Conjugated Gelatin Integrated with Genipin and Epigallocatechin Gallate
Source: Gels. 2026 Jan 11;12(1):67. doi: 10.3390/gels12010067 (PMC12840651; doi:10.3390/gels12010067)
Supplement: Supplementary file 1 [file gels-12-00067-s001.zip › gels-4012089-supplementary.pdf]

Supporting Information

Biomedical Interpenetrated Hydrogels Fabricated via Quaternary Ammonium Chitosan and Dopamine-Conjugated Gelatin Integrated with Genipin and Epigallocatechin Gallate

Ling Wang [1], Shuxin Hu [1], Zheng Wei [1], Peng Ding [1], Yaling Deng [2], Yanting Han [1], Yanfang Sun [3], Guohua Jiang [4,5] and Lei Nie [1,*]

[1] College of Life Sciences, Xinyang Normal University (XYNU), Xinyang 464000, China; wangling@xynu.edu.cn (L.W.); hushuxin060907@163.com (S.H.); weizheng20050815@163.com (Z.W.); dingzhiyu120@163.com (P.D.); hanyt@xynu.edu.cn (Y.H.)
[2] College of Intelligent Science and Control Engineering, Jinling Institute of Technology, Nanjing 211169, China; yalingdeng@jit.edu.cn
[3] College of Life Sciences and Medicine, Zhejiang Sci-Tech University, Hangzhou 310018, China; katherineyfs@zstu.edu.cn
[4] School of Materials Science and Engineering, Zhejiang Sci-Tech University, Hangzhou 310018, China; ghjiang_cn@zstu.edu.cn
[5] International Scientific and Technological Cooperation Base of Intelligent Biomaterials and Functional Fibers, Zhejiang Sci-Tech University, Hangzhou 310018, China
* Correspondence: nielei@xynu.edu.cn or nieleifu@yahoo.com; Tel: +86-13600621068

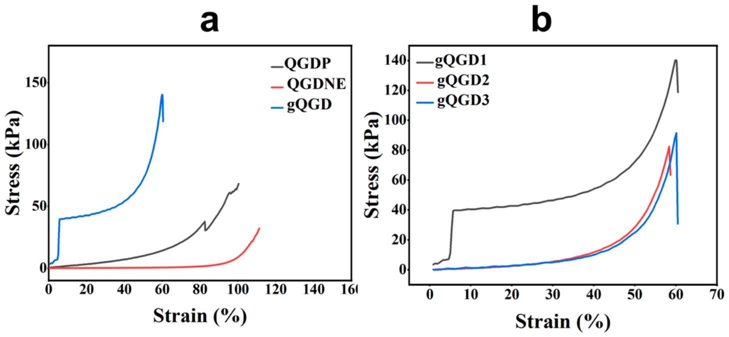

Figure S1. (a) Stress-strain curves of QGDP, QGDNE, and gQGD hydrogels during the tensile strength test. (b) Stress-strain curves of the gQGD hydrogels.

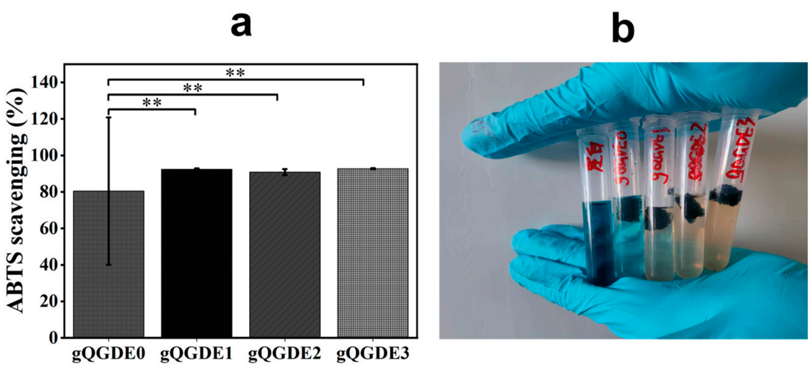

Figure S2. (a) ABTS scavenging rates of the gQGD hydrogels, *$p < 0.05$. (b) Photos recorded display the hydrogels immersed in ABTS solution during the ABTS scavenging test.

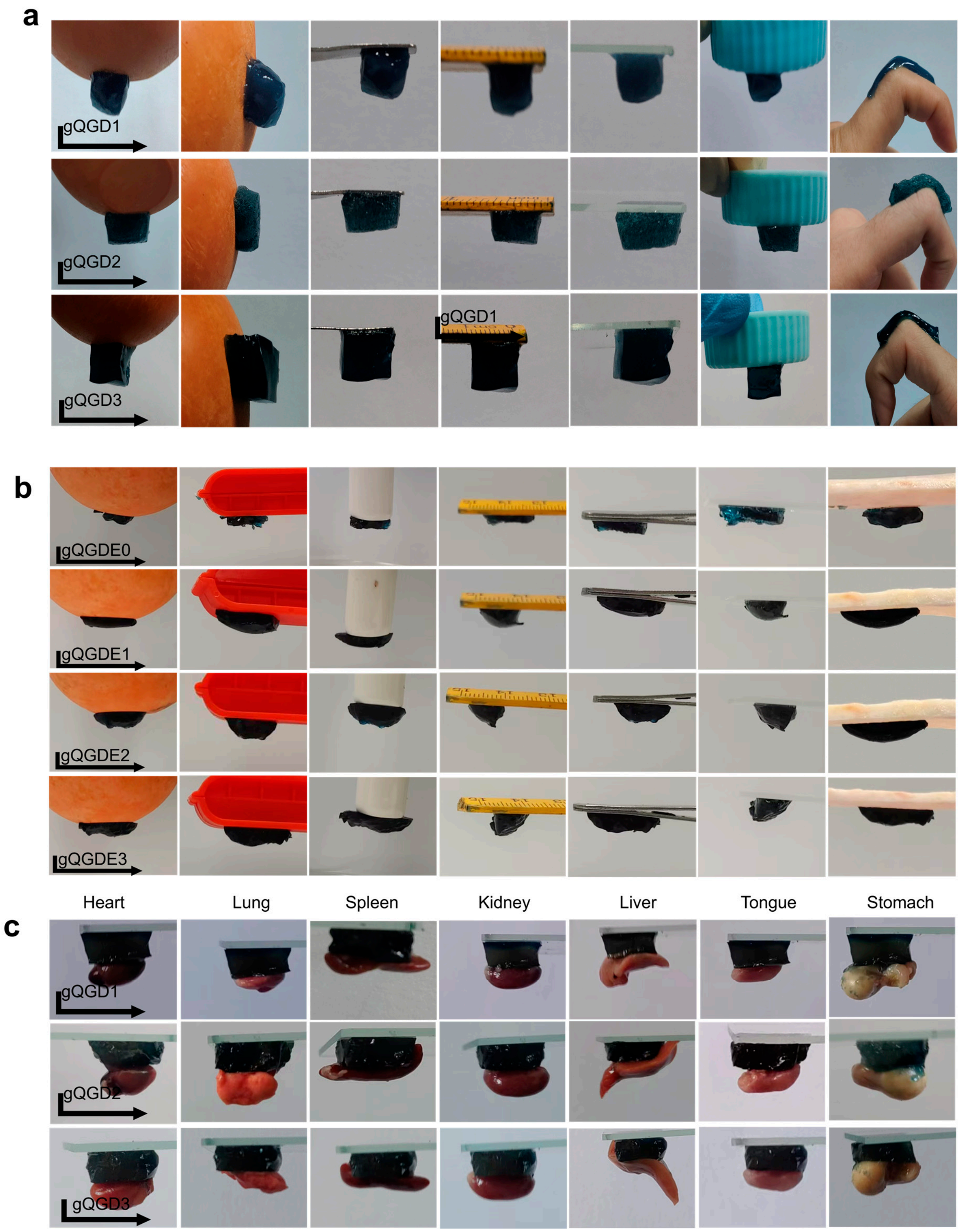

Figure S3. (a) The fabricated gQGD hydrogels could firmly adhere on different matrix surface, including rubber, plastic, ceramics, wood, metal, glass, skin, and rubber gloves. (b) The fabricated gQGDE hydrogels exhibit good adhesive performance, the hydrogels could firmly adhere on the surface of different matrix, including rubber, plastic, ceramics, wood, metal, glass and pig skin. (c) The gQGD hydrogels exhibit good adhesive performance on different organs, including heart, lung, spleen, kidney, liver, tongue, and stomach.
